# Using site-directed mutagenesis to probe the role of the D2 carotenoid in the secondary electron-transfer pathway of photosystem II

**DOI:** 10.1007/s11120-013-9793-6

**Published:** 2013-01-21

**Authors:** Katherine E. Shinopoulos, Jianfeng Yu, Peter J. Nixon, Gary W. Brudvig

**Affiliations:** 1 Department of Chemistry, Yale University, New Haven, CT 06520-8107 USA; 2 Division of Molecular Biosciences, Sir Ernst Chain Building – Wolfson Laboratories, Imperial College London, S. Kensington campus, London, SW7 2AY UK

**Keywords:** β-Carotene radical, Chlorophyll radical, Cytochrome *b*_559_, EPR spectroscopy, Near-IR spectroscopy, Photosystem II, Site-directed mutagenesis

## Abstract

Secondary electron transfer in photosystem II (PSII), which occurs when water oxidation is inhibited, involves redox-active carotenoids (Car), as well as chlorophylls (Chl), and cytochrome *b*_559_ (Cyt *b*_559_), and is believed to play a role in photoprotection. Car_D2_ may be the initial point of secondary electron transfer because it is the closest cofactor to both P_680_, the initial oxidant, and to Cyt *b*_559_, the terminal secondary electron donor within PSII. In order to characterize the role of Car_D2_ and to determine the effects of perturbing Car_D2_ on both the electron-transfer events and on the identity of the redox-active cofactors, it is necessary to vary the properties of Car_D2_ selectively without affecting the ten other Car per PSII. To this end, site-directed mutations around the binding pocket of Car_D2_ (D2-G47W, D2-G47F, and D2-T50F) have been generated in *Synechocystis* sp. PCC 6803. Characterization by near-IR and EPR spectroscopy provides the first experimental evidence that Car_D2_ is one of the redox-active carotenoids in PSII. There is a specific perturbation of the Car^∙+^ near-IR spectrum in all three mutated PSII samples, allowing the assignment of the spectral signature of Car_D2_^∙+^; Car_D2_^∙+^ exhibits a near-IR peak at 980 nm and is the predominant secondary donor oxidized in a charge separation at low temperature in ferricyanide-treated wild-type PSII. The yield of secondary donor radicals is substantially decreased in PSII complexes isolated from each mutant. In addition, the kinetics of radical formation are altered in the mutated PSII samples. These results are consistent with oxidation of Car_D2_ being the initial step in secondary electron transfer. Furthermore, normal light levels during mutant cell growth perturb the shape of the Chl^∙+^ near-IR absorption peak and generate a dark-stable radical observable in the EPR spectra, indicating a higher susceptibility to photodamage further linking the secondary electron-transfer pathway to photoprotection.

**Electronic supplementary material:**

The online version of this article (doi:10.1007/s11120-013-9793-6) contains supplementary material, which is available to authorized users.

## Introduction

**Fig. 1:**
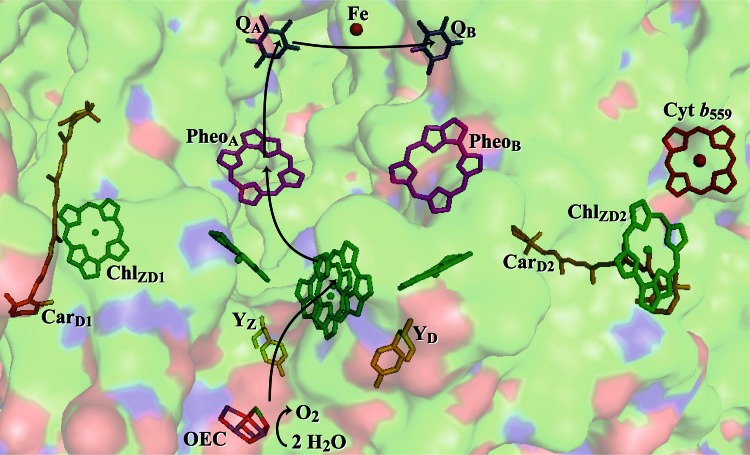
The arrangement of cofactors in the D1/D2/Cyt *b*_559_ sub-complex of cyanobacterial PSII, viewed along the membrane plane (PDB ID: 3ARC). *Black arrows* represent electron transfer. The oxygen-evolving complex (OEC) is shown with manganese ions in *purple*, oxygen in *red*, and calcium in *green*; tyrosine Z (Y_Z_) and tyrosine D (Y_D_) are shown in *yellow*; chlorophylls (Chl) are shown in *green*; β-carotenes (Car) are shown in *orange*; pheophytins (Pheo_A_ and Pheo_B_) are shown in *magenta*; quinones (Q_A_ and Q_B_) are shown in *blue*; and cytochrome *b*_559_ (Cyt *b*_559_) and the nonheme iron are shown in *red*. The surface of the protein is shown in the background and colored according to atom identity with C in *green*, N in *blue*, and O in *red*

Photosystem II (PSII) is the enzyme responsible for photosyntheic oxidation of water to O_2_, generating the reducing equivalents that ultimately are used for CO_2_ fixation. Water oxidation occurs when excitons are transferred to a special group of chlorophylls, known as P_680_, where a charge separation occurs, as seen in Fig. 1. The electron is transferred to Pheo_A_ on a timescale of tens of picoseconds (Holzwarth et al. 2006), and then to Q_A_ with a timescale of 200–500 picoseconds (ps) (Rappaport and Diner 2008). The electron–hole pair on P_680_^+^ and Q_A_^−^ is stable for close to 1 ms in cyanobacteria (Reinman et al. 1981; Gerken et al. 1989; Metz et al. 1989), during which time, under catalytic conditions, the oxygen-evolving complex (OEC) donates an electron to P_680_^+^ via a redox-active tyrosine, Y_Z_. Once the OEC, which consists of a Mn_4_CaO_5_ cluster (Umena et al. 2011), has been oxidized four times via sequential charge separations to reach a high-valent state, probably Mn(IV)Mn(IV)Mn(IV)Mn(IV)-O^∙^ (Siegbahn 2006; Sproviero et al. 2008), it is capable of oxidizing water to dioxygen. Meanwhile, the electron on Q_A_ is transferred to Q_B_, which dissociates away from PSII after two reductions and subsequent protonations, carrying reducing equivalents to the next step in photosynthesis and ultimately resulting in the storage of energy in the chemical bonds of sugars.

However, the intermediates associated with water splitting are very oxidizing, and cause damage to the protein over time. The D1 subunit of PSII, which contains most of the cofactors involved in water oxidation, turns over every 30 min, in a process that involves disassembly of the PSII complex, membrane diffusion, and protein synthesis (Nixon et al. 2010). In order to minimize damage, PSII has evolved multiple mechanisms of photoprotection to prolong the lifetime of its subunits and minimize energy expenditure for protein synthesis. One mechanism involves adjusting the size of the light-harvesting antenna; other mechanisms involve dissipating excess solar energy as heat, as in the xanthophyll cycle in plants (Niyogi 1999) or via the orange carotenoid protein in cyanobacteria (Kirilovsky and Kerfeld 2012). In addition, when water-oxidation catalysis is impaired, oxidation of secondary donors, including carotenoids (Car), chlorophylls (Chl), and cytochrome *b*_559_ (Cyt *b*_559_), may serve to remove excess oxidizing equivalents from PSII (Thompson and Brudvig 1988; Buser et al. 1992) or to quench chlorophyll excited states (Schweitzer and Brudvig 1997).

Of the secondary electron donors, Cyt *b*_559_ has the lowest reduction potential, which is interestingly variable from 390 mV to as low as −150 mV (Thompson et al. 1989; Stewart and Brudvig 1998). Cyt *b*_559_ is, therefore, the terminal secondary electron donor within PSII. It may additionally be rereduced by the plastoquinone pool, leading to a cyclic process for the removal of excess, damaging oxidizing equivalents from PSII when the system is unable to drive water oxidation (Shinopoulos and Brudvig 2012).

**Fig. 2:**
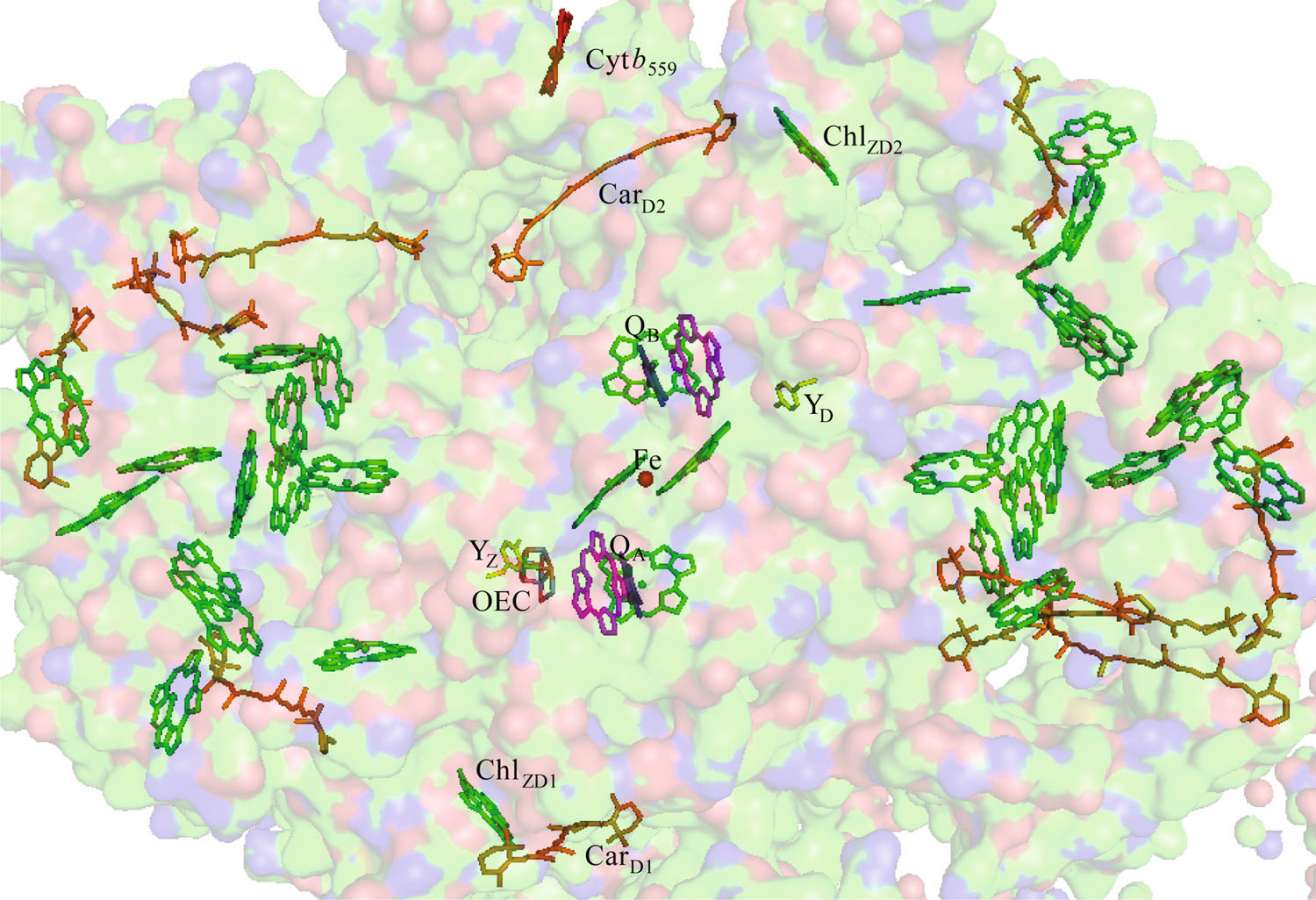
The arrangement of cofactors in PSII, viewed from the membrane surface (PDB ID: 3ARC). The oxygen-evolving complex (OEC) is shown with manganese atoms in *purple*, oxygen in *red*, and calcium in *green*; tyrosine Z (Y_Z_) and tyrosine D (Y_D_) are shown in *yellow*; chlorophylls (Chl) are shown in *green*; β-carotenes (Car) are shown in *orange*; pheophytins (Pheo_A_ and Pheo_B_) are shown in *magenta*; quinones (Q_A_ and Q_B_) are shown in *blue*; and cytochrome *b*_559_ (Cyt *b*_559_) and the nonheme iron are shown in *red*. The surface of the protein is shown in the background and colored according to atom identity with C in *green*, N in *blue*, and O in *red*

Although the final location of the oxidizing equivalent passed along the secondary electron-transfer pathway has been determined to be Cyt *b*_559_ (Vermeglio and Mathis 1974; de Paula et al. 1985), the pathway of electron transfer from Cyt *b*_559_ to P_680_^+^ has not been fully characterized. The distance of about 40 Å between the two cofactors indicates that they do not participate in direct electron transfer, and it has indeed been observed that Chl and Car are intermediates (de Paula et al. 1985; Hanley et al. 1999; Vrettos et al. 1999; Tracewell et al. 2001; Faller et al. 2001). It has also been shown that there are at least two redox-active carotenoids (Car^∙+^) in PSII based on the shift of the Car^∙**+**^ near-IR peak over a range of illumination temperatures and the wavelength-dependant decay rate of the Car^∙**+**^ absorbance (Tracewell and Brudvig 2003; Telfer et al. 2003). There are as many as 5 redox-active Chl (Chl^∙+^) (Tracewell and Brudvig 2008; Telfer et al. 1990), with one ligated to D1-His 118 (Stewart et al. 1998). However, there are 11 Car and 35 Chl per PSII, as seen in Fig. 2, and most of the redox-active cofactors have not been specifically identified. Some Chl^∙+^ may be in CP43 and CP47, peripheral subunits that bind many Chl molecules (Tracewell and Brudvig 2008). In regard to the two Car^∙+^, it has been observed that the average distance from the nonheme iron to the two Car^∙+^ is 38 Å, and it has been hypothesized that one Car^∙+^ is Car_D2_^∙+^ (Lakshmi et al. 2003; Tracewell and Brudvig 2003). This seems likely, because Car_D2_ is the closest cofactor to both P_680_ and Cyt *b*_559_, with edge-to-edge distances of 11 and 12 Å, respectively. The oxidation of Y_D_ results in a shift of the Car^∙+^ near-IR peak, indicating proximity of at least one Car^∙+^ to Y_D_ (Tracewell and Brudvig 2003), although electrochromic effects can propagate significant distances though PSII (Stewart et al. 2000). A relatively higher yield of Car^∙+^ than Chl^∙+^ is observed at lower temperatures, with increased Chl^∙+^ at higher temperatures, also indicating that Car^∙+^ is closer than Chl^∙+^ to P_680_ (Hanley et al. 1999).

**Fig. 3:**
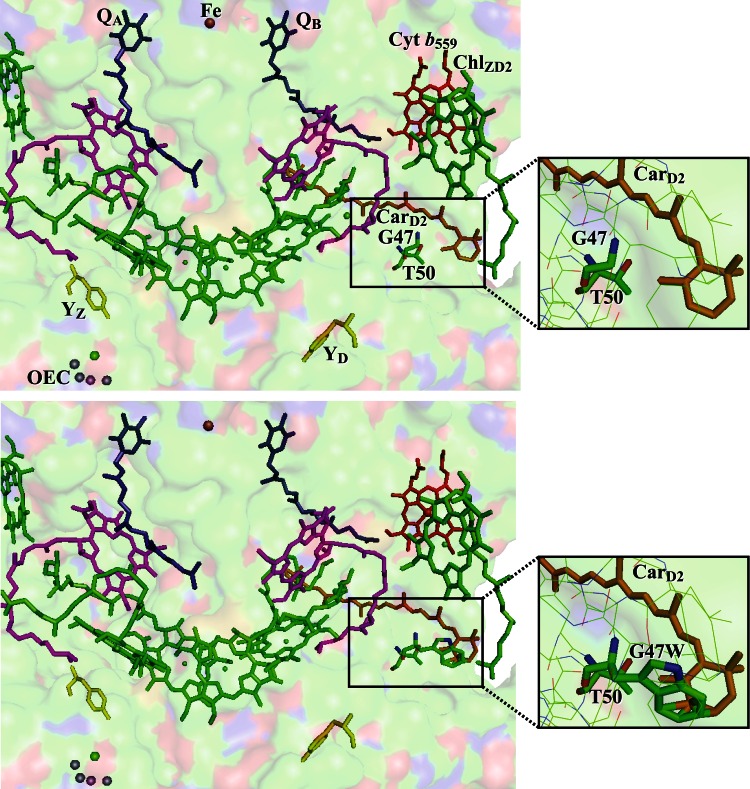
Electron-transfer cofactors in photosystem II, viewed along the membrane plane (PDB ID: 2AXT). The oxygen-evolving complex (OEC) is shown with manganese atoms in *purple* and calcium in *green*; tyrosine Z (Y_Z_) and tyrosine D (Y_D_) are shown in *yellow*; chlorophylls (Chl) are shown in *green*; β-carotene (Car) is shown in *orange*; pheophytins (Pheo_A_ and Pheo_B_) are shown in *magenta*; quinones (Q_A_ and Q_B_) are shown in *blue*; and cytochrome *b*_559_ (Cyt *b*_559_) and the nonheme iron are shown in *red*. The surface of the protein is shown in the background and colored according to atom identity with C in *green*, N in *blue*, and O in *red*. **Top** A model of WT PSII structure, containing D2-G47 and D2-T50 modeled in stick form. *Inset* an enlarged picture of G47, T50, and the β-ionylidene ring of Car_D2_ with the surrounding residues shown as *lines*, colored according to atom identity. **Bottom** A model of D2-G47W, with G47W and T50 modeled in stick form. *Inset* an enlarged picture of G47W, T50, and the β-ionylidene ring of Car_D2_ with the surrounding residues shown as *lines*, colored according to atom identity

In order to evaluate the role of Car_D2_ in secondary electron transfer relative to the roles of other Car in PSII, we have characterized the effects of site-directed mutations around the binding pocket of Car_D2_ (see Fig. 3). In this study, the effects of the mutations D2-G47W, D2-T50F, and D2-G47F on the secondary electron-transfer pathway are examined by low temperature near-IR optical and EPR spectroscopy.

## Materials and methods

### Chemicals and reagents

2-(*N*-morpholino)-ethanesulfonic acid (MES) was purchased from USB Corporation. β–Dodecyl maltoside (β-DM) was purchased from Enzo Life Sciences International Inc. A stock solution (80 mM) of potassium ferricyanide (purchased from Sigma-Aldrich) was prepared in buffer and frozen until use.

### Mutagenesis

D2 mutants were constructed according to (Tang et al. 1993) except that the recipient strain Tol145/CP47-His, obtained by transforming strain Tol145 (Tang et al. 1993) with genomic DNA from strain PSII-His (Boehm et al. 2011), also encoded a C-terminal His-tagged derivative of CP47. Plasmid pDC074 was used as the parental vector for site-directed mutagenesis (Tang et al. 1993). Mutations were introduced into the plasmid by overlap-extension PCR so that the codon specifying D2-G47 was replaced by either TGG (to make mutated D2-G47W) or TTC (D2-G47F) and the codon specifying D2-T50 was replaced by TTC (D2-T50F). In all three cases, the codon for Leu45 (CTG) was mutated to incorporate a silent mutation (CTA), in order to create a unique restriction site, AvrII, to help screen for mutations. The genotypes of the cyanobacterial mutants were confirmed by PCR analysis and DNA sequencing.

### Cell growth and protein purification

Cells were grown initially on plates containing 5 mM glucose, 10 μM DCMU, 25 mg/L kanamycin, and 10 mg/L erythromycin. In liquid culture, the cells were grown without antibiotics in the presence of 5 mM glucose under 10 or 40 μEinsteins/m^2^/s of illumination, as noted. His-tagged PSII core particles were isolated from *Synechocystis* PCC 6803 cells as previously described (Lakshmi et al. 2002).

### Sample treatments

For low-temperature measurements, PSII samples were transferred to a buffer containing 15 mM CaCl_2_, 63 % (v/v) glycerol, and 50 mM MES at pH 6.0. Prior to freezing, PSII samples were treated with 5 mM ferricyanide to oxidize Cyt *b*_559_.

### Near-IR optical spectroscopy

A Perkin-Elmer Lambda 20 spectrometer was used to make optical spectroscopic measurements in the visible and near-IR. Low-temperature optical measurements were made with an Oxford Instruments Optistat liquid helium cryostat. Polyethylene cuvettes with a 1.0 cm path length and 0.4 cm width (Fisher Scientific) were used for low-temperature optical measurements. A 150 W quartz-halogen lamp filtered by a 6 in water filter and a heat-absorbing filter (Schott KG-5) was used to illuminate samples. A Schott-Fostec randomized fiber optic bundle was used to direct the light into the cryostat. The PSII samples were prepared as previously described (Tracewell and Brudvig 2008). Illumination for 15 min was performed on samples that were equilibrated at the specified temperature for at least 60 min in the cryostat. All spectra collected after illumination are referenced to the dark spectrum measured at the same temperature to avoid contributions from spectral changes in the background due to temperature effects.

### Spectral simulations

The program Igor Pro 6.2 was used to simulate the near-IR absorption data, to analyze the decay kinetics, and to plot all spectra.

### EPR spectroscopy

X-band EPR measurements were conducted on a Bruker ELEXSYS E500 EPR spectrometer equipped with an Oxford ESR 900 He-flow cryostat and a Super High Q cavity. Samples were illuminated by a xenon halogen lamp filtered by a 6 in water filter and a heat-absorbing filter, with a fiber optic cable directing light into the cryostat. Radical yields per PSII were determined by integration of the derivative EPR signals and calibrated to photooxidized tyrosine D (Y_D_^•^). Y_D_^•^ was generated by illuminating the PSII samples for 30 s at 0 °C, incubating on ice for 2 min, and freezing in total darkness.

## Results

### Selection of mutations

The mutations D2-G47F, D2-G47W, and D2-T50F were selected by using Coot, a modeling program that includes the ability to mutate a selected residue from a known crystal structure (Emsley and Cowtan 2004). The mutated residue is placed in the conformation in which it is typically found, and other conformations are also observable. Using the 3.0-Å resolution crystal structure of PSII (Loll et al. 2005), which was focused on accurate cofactor positioning and found similar locations to the recent 1.9-Å resolution structure (Umena et al. 2011), residues that would sterically interfere with Car_D2_ binding were identified, as shown in Fig. 3. Aromatic residues have been observed around the β-ionylidene ring binding site (Tracewell and Brudvig 2003), which has been found to be important for function (Bautista et al. 2005), and because the Car chain exists in a variety of conformations in PSII samples, the area near the rings was targeted for mutation. In this way, several mutations were identified that may cause a disruption to the hydrophobic binding pocket of the β-ionylidene ring of Car_D2_.

### Near-IR Optical Spectroscopy

**Fig. 4:**
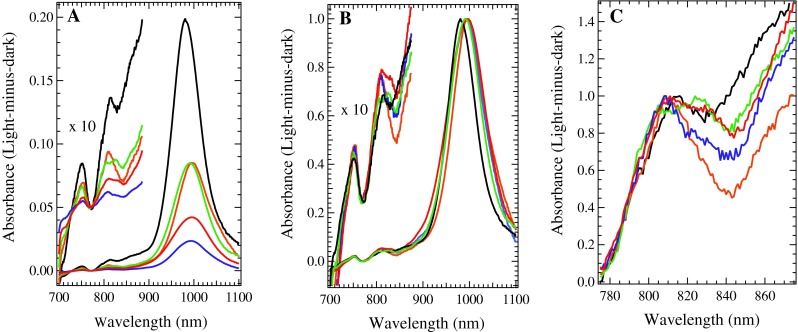
Light-minus-dark near-IR spectra of *Synechocystis* PSII samples from WT cells grown under 40 μEinsteins/m^2^/s of illumination (*black*), T50F cells grown under 10 μEinsteins/m^2^/s of illumination (*green*), T50F cells grown under 40 μEinsteins/m^2^/s (*orange*), G47W cells grown under 10 μEinsteins/m^2^/s of illumination (*red*), and G47F cells grown under 40 μEinsteins/m^2^/s of illumination (*blue*), recorded at 20 K. **A** Normalized to equal chlorophyll concentration. **B** Each Car^∙+^ peak normalized to 1. **C** Each Chl^∙+^ peak normalized to 1

WT, D2-T50F, D2-G47W, and D2-G47F His-tagged PSII complexes were illuminated in a cryostat at 20 K for 15 min, maximally generating one stable charge separation per PSII center; at this temperature in ferricyanide-treated samples, the stable charge separation results in an electron on Q_A_^−^ and a hole that is located on either a Car neutral radical (Car^∙^ absorbing at 750 nm), a Chl cation radical (Chl^∙+^ absorbing at 800–840 nm) or a Car cation radical (Car^∙+^ absorbing near 1,000 nm), as seen in Fig. 4. For each mutated PSII sample, the total yield of stable charge separated states was lower than in WT PSII samples when normalized to the same concentration of Chl, indicated by the lower yield of all secondary donors (Car^∙^, Chl^∙+^, and Car^∙+^), seen in Fig. 4A. When the magnitudes of the Car^∙+^ peaks are normalized to 1, as in Fig. 4B, it can be seen that the Car^∙+^ peak is slightly red shifted and has a larger FWHM in mutated PSII samples compared to WT PSII samples. The yield of the Car^∙^ peak at 750 nm tracks with the magnitude of the Car^∙+^ peak, reinforcing that it is generated from Car (Gao et al. 2009). In the mutated PSII samples, there is slightly more Chl^∙+^ generated relative to Car^∙+^ than in WT, especially in the G47F and G47W PSII samples, with an absorbance centered at 825 nm. Although the yield of Chl^∙+^ appears to be very low, it has an extinction coefficient of about 7,000 M^−1^ cm^−1^ (Borg et al. 1970), while Car^∙+^ has an extinction coefficient of about 160,000 M^−1^ cm^−1^ (Tan et al. 1997). The width and shape of the Chl^∙+^ peak varies among the samples, as seen in Fig. 4C. The T50F PSII sample isolated from cells grown at 10 μEinsteins/m^2^/s of illumination has the narrowest peak, followed closely by G47F PSII samples. PSII samples isolated from G47W, T50F grown under 40 μEinsteins/m^2^/s of illumination, and WT cells display wider Chl^∙+^ signatures that appear to contain two peaks.

Using global analysis in Igor Pro 6.2, the Car^∙+^ peak in all PSII samples was deconvoluted into two Gaussian contributions. One contribution had a maximum at 999–1,003 nm, while the other varied from 980 nm in WT PSII to 993 nm in G47W PSII, as seen in Table 1. The FWHM of the Gaussian components were, in general, larger in the mutated PSII samples, with the widest peaks appearing in the G47 W PSII spectrum.

**Table 1:** The peak parameters of the two Gaussian components of the Car^∙+^ peak present in WT, T50F, G47F, and G47W PSII samples

	λ_1_ (nm)	Initial %	FWHM_1_ (nm)	λ_2_ (nm)	Initial %	FWHM_2_ (nm)
WT	980.4	69	37.9	999.2	31	74.1
T50F	989.3	68	43.2	999.8	32	92.8
G47F	988.3	48	40.8	1001	52	68.0
G47W	993.3	82	55.0	1003	17	127

**Fig. 5:**
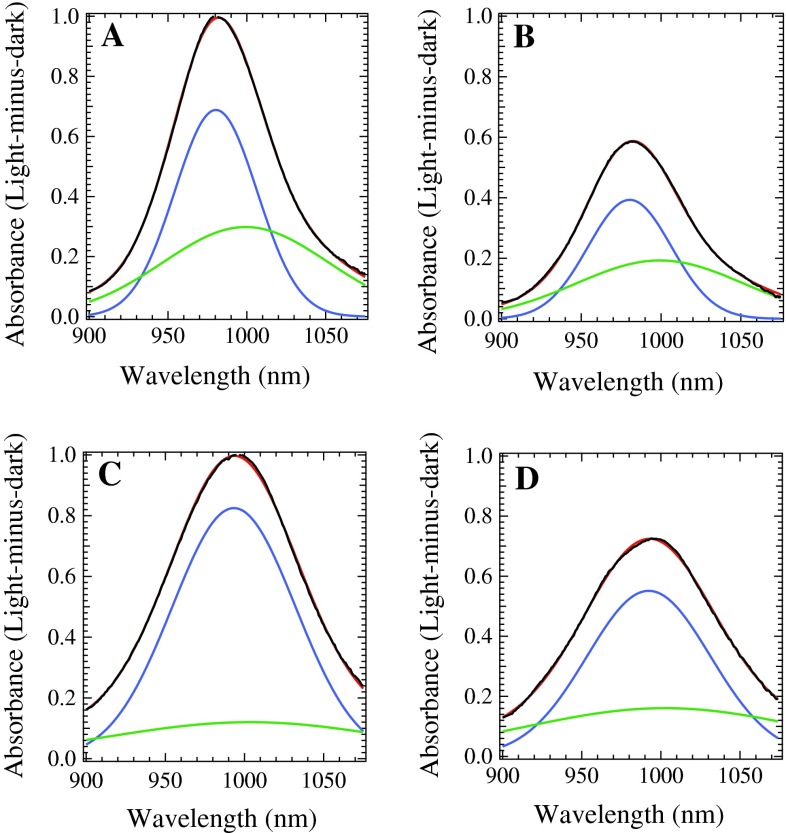
Gaussian deconvolutions of the Car^∙+^ peak formed by illumination for 15 min at 20 K. **A** The WT PSII difference spectrum after 0 min of dark incubation. **B** The WT PSII difference spectrum after 3 h of dark incubation. **C** The G47W PSII difference spectrum after 0 min of dark incubation. **D** The G47W PSII difference spectrum after 3 h of dark incubation. The two Gaussian components from Table 1 are shown in blue (shorter-wavelength component) and *green* (longer-wavelength component), their sum is shown in *red*, and the raw data are shown in *black*

**Fig. 6:**
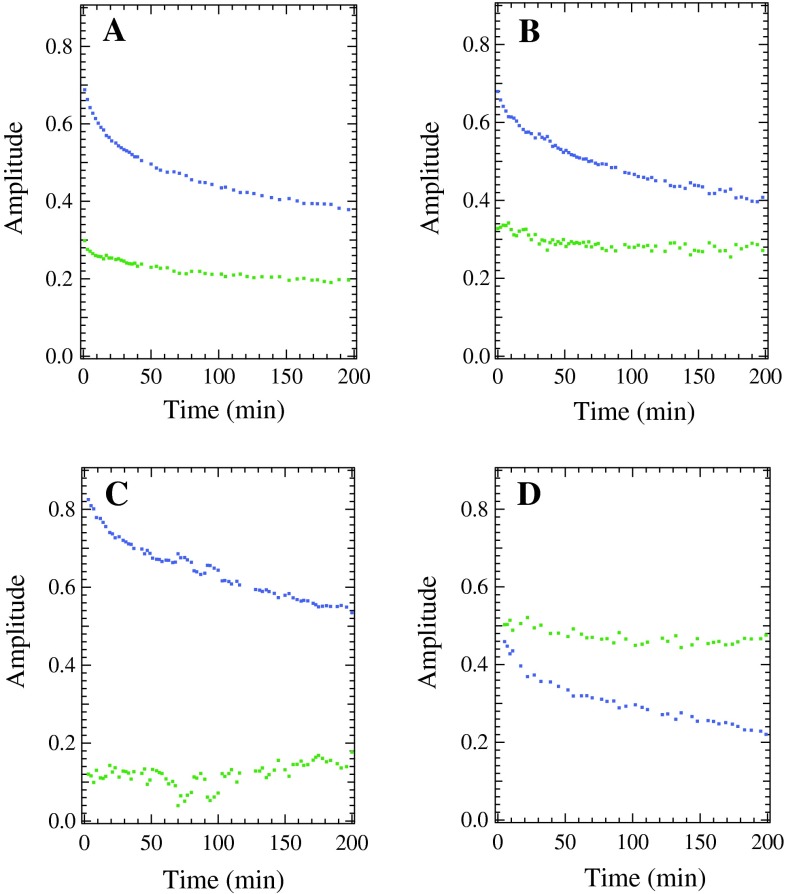
The decay in absorbance, as a function of dark incubation time, of the shorter-wavelength component (*blue*) and the longer-wavelength component (*green*). **A** WT PSII samples. **B** T50F PSII samples. **C** G47W PSII samples. **D** G47F PSII samples

The relative amounts of the longer-wavelength component and shorter-wavelength component varied among the WT and mutated PSII samples, with the G47F PSII spectrum containing the most longer-wavelength component, the G47W spectrum containing the least longer-wavelength component, and the WT and T50F spectra containing a similar ratio to each other, as seen in Table 1; Figs. 5 and 6. In addition, in each PSII sample, the shorter-wavelength component of the Car^∙+^ peak decayed more quickly and to a larger extent. Therefore, there was a larger proportion of the longer-wavelength component present at longer times.

### EPR Spectroscopy

**Fig. 7:**
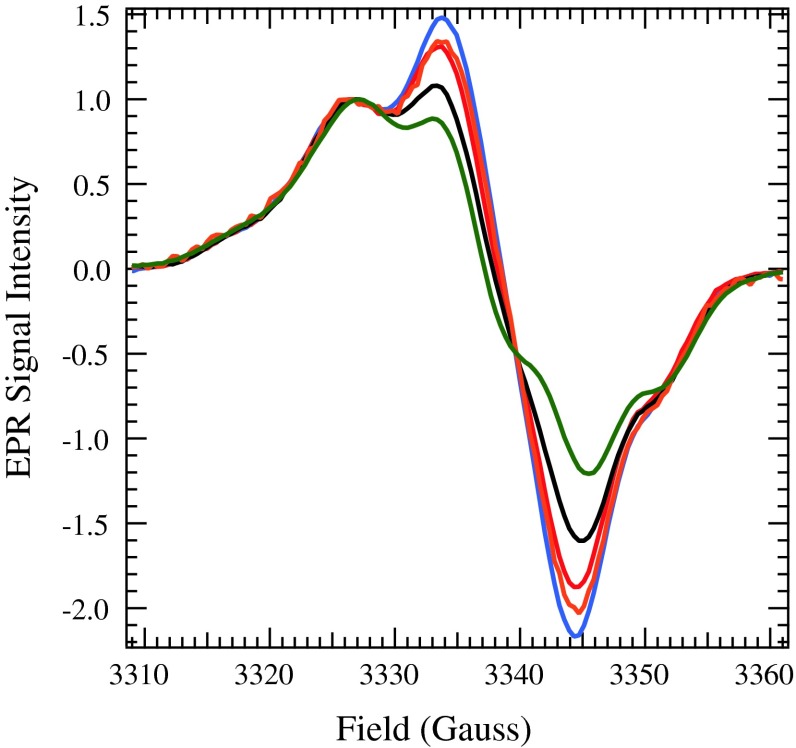
EPR spectra in the Y_D_^∙^ region of PSII isolated from WT cells grown under 40 μEinsteins/m^2^/s of illumination (*black*), T50F cells grown under 10 μEinsteins/m^2^/s of illumination (*green*), T50F cells grown under 40 μEinsteins/m^2^/s (*orange*), G47W cells grown under 40 μEinsteins/m^2^/s of illumination (*red*), and G47F cells grown under 40 μEinsteins/m^2^/s of illumination (*blue*). Instrument settings:  temperature, 30 K; microwave power, 105 μW; and field modulation amplitude, 4 G

Following the generation of Y_D_^∙^, EPR spectra of WT, D2-T50F, D2-G47W, and D2-G47F PSII samples were collected in total darkness at 30 K, as seen in Fig. 7. The lineshapes vary slightly among the spectra. The spectra of T50F PSII grown at 10 μEinsteins/m^2^/s of illumination exhibit the most characteristic Y_D_^∙^ pattern. The WT spectrum also matches the lineshape reported in the literature for Y_D_^∙^ (Un et al. 1996; Tang et al. 1993; Noren et al. 1991). However, the spectra of PSII isolated from G47 W, T50F grown at 40 μEinsteins/m^2^/s of illumination, and G47F cells deviate increasingly from a normal Y_D_^∙^ spectrum. The shift could be due to a change in the orientation of the methylene protons with respect to the ring in the tyrosyl radicals of the mutated PSII samples, resulting in altered hyperfine coupling (Barry and Babcock 1998). However, due to the shift in *g* value of the baseline crossing point toward the free-electron *g* value and the consistency of the most upfield and downfield hyperfine peaks, it appears that the change in lineshape is due to an organic radical signal overlapping with Y_D_^∙^. Although this is consistent with the presence of Chl^∙+^ and Car^∙+^, which may be generated by illumination, these species have a very short lifetime at 0 °C, and would have typically decayed during dark incubation. In addition, there is a larger amount of the organic radical signature present in the spectrum from T50F grown at 40 μEinsteins/m^2^/s of illumination than is present in the spectrum from T50F grown at 10 μEinsteins/m^2^/s of illumination, indicating that the presence of an overlapping radical EPR signal is due to an effect of high light during growth of the cells rather than an effect of the mutation on the structure of Y_D_^∙^.

**Fig. 8:**
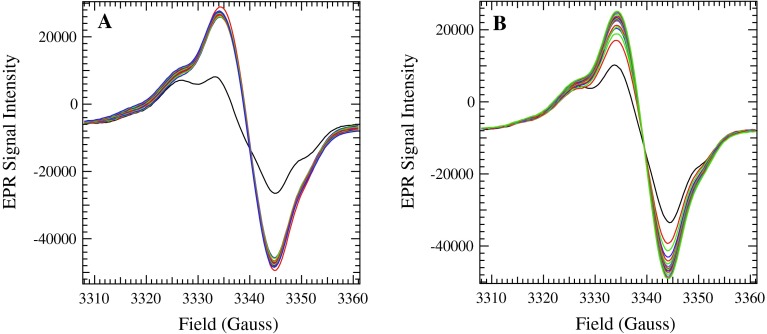
The EPR spectra collected as samples were illuminated in the cryostat with a xenon lamp for 1 h. **A** WT spectra collected in the dark (*black*) and after 0 (*red*), 5 (*green*), 10 (*blue*), 15 (*red*), 20 (*green*), 25 (*blue*), 30 (*blue*), 35 (*red*), 40 (*green*), 45 (*blue*), 50 (*red*), 55 (*green*), and 60 (*blue*) minutes of illumination. **B** G47F spectra collected in the dark (*black*) and after 2 (*red*), 8 (*green*), 12 (*blue*), 17 (*red*), 22 (*green*), 25 (*blue*), 30 (*red*), 34 (*green*), 38 (*blue*), 42 (*red*), 47 (*green*), 51 (*blue*), 55 (*red*), and 60 (*green*) minutes of illumination. Instrument settings as in Fig. 7

**Fig. 9:**
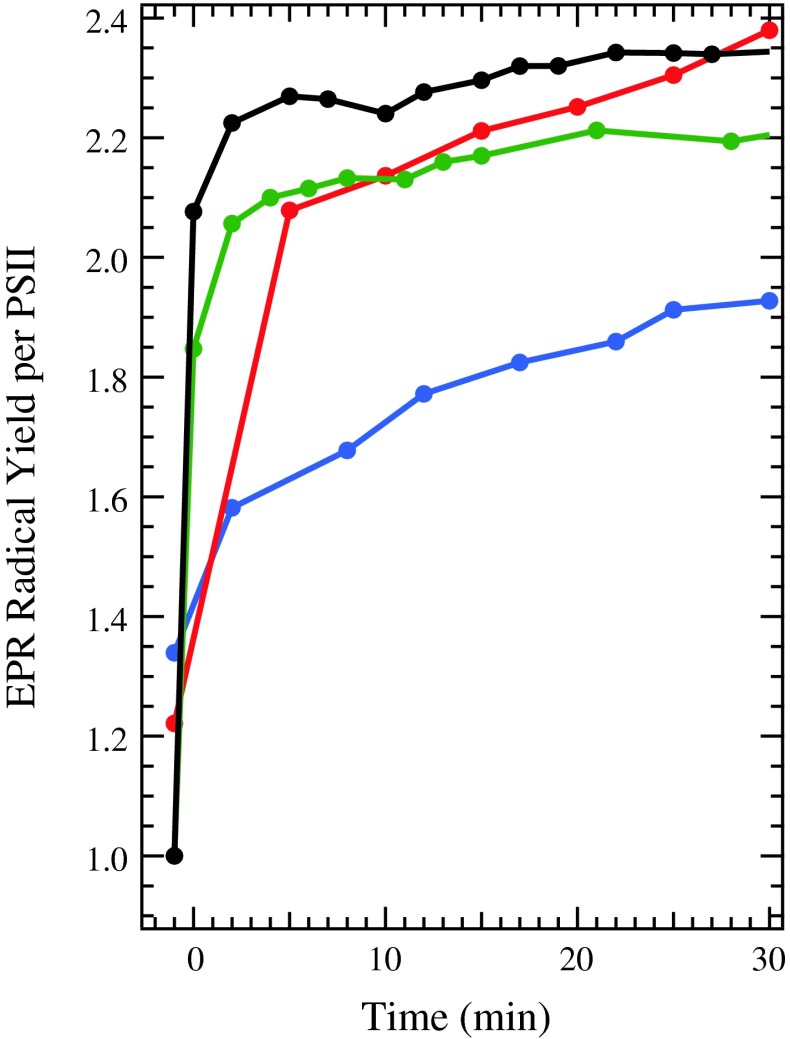
The radical yield per PSII as a function of illumination time, obtained by double integration of the EPR spectra of WT (*black*), T50F (*green*), G47W (*red*), and G47F (*blue*) PSII samples, recorded at 30 K. Instrument settings as in Fig. 7

The samples containing Y_D_^∙^ were subsequently illuminated in the cryostat at 30 K for 60 min and spectra were recorded during the illumination, as seen in Figs. 8 and 9. During the illumination, Chl^∙+^ and Car^∙+^ (Figs. 8 and 9), which have indistinguishable *g* values at X band (Hanley et al. 1999), and some oxidized Cyt *b*_559_ (data not shown) were formed. For the WT PSII sample (Fig. 8A), the total yield of oxidized secondary donors was generated within 5 min of illumination. In contrast, in the G47F PSII sample (Fig. 8B), the maximum yield of oxidized secondary donors was not reached until after 30 min of illumination.

The procedure for generating Y_D_^∙^ results in one dark-stable Y_D_^∙^ per PSII, formed on ice before the sample is frozen for measurement of the EPR spectrum. Double integration of the Y_D_^∙^ EPR spectra gives the area corresponding to one radical per PSII center, once it has been corrected for any other overlapping organic radical signals. Under illumination at cryogenic temperatures, PSII is limited to one charge separation, and a second EPR signal is generated from the electron donor in each PSII center. Therefore, there should be a total of two oxidized species on the electron donor side of PSII for each PSII center: one Y_D_^∙^ and either Chl^∙+^, Car^∙^, Car^∙+^, or oxidized Cyt *b*_559_. However, the kinetics of formation of the second radical varied among the WT and mutated PSII samples, as seen in Fig. 9. WT and T50F samples generated the full radical yield within a few minutes of illumination. G47W samples took slightly longer to reach the total yield, while G47F samples reached two radicals per PSII only after a full 60 min of illumination (data from 30 to 60 min not shown).

## Discussion

Car_D2_ occupies a position between P_680_, the initial oxidant, and Cyt *b*_559_, the terminal electron donor, in the path of secondary electron transfer. Removing or disrupting this cofactor would be expected to alter the electron-transfer properties of PSII, if Car_D2_ is involved as an early donor in the secondary electron-transfer pathway. Indeed, the yields and kinetics of Car and Chl radical formation are altered in PSII samples that have been mutated to alter D2-G47 and D2-T50, two amino acids near Car_D2_. We have studied the properties of these mutated PSII complexes in which Car_D2_ is perturbed in order to gain more information on the secondary electron-transfer cofactors and their connectivity.

At cryogenic temperatures, illumination generates one stable charge separation per PSII, resulting in the formation of Q_A_^−^ and either Car^∙^, Chl^∙+^, Car^∙+^, or oxidized Cyt *b*_559_. Cyt *b*_559_, which has the lowest reduction potential, is the preferred and terminal secondary electron donor within PSII. When Cyt *b*_559_ is preoxidized, one Car^∙^, Chl^∙+^, or Car^∙+^ intermediate is observed per PSII center upon illumination. The relative amounts of these radicals generated in a PSII sample are affected by temperature (Tracewell and Brudvig 2003) and by sample conditions (Hanley et al. 1999). If there were one accessible cofactor with the lowest reduction potential in each PSII center, a single radical would be generated, rather than a distribution. Therefore, the cofactors must be closely spaced in redox potential and have good connectivity to result in different radicals being trapped in different PSII centers. The D2-G47W, D2-G47F, and D2-T50F mutations are located near the headgroup of Car_D2_ and are expected to perturb Car_D2_ sterically, while the F and W residues may also participate in π stacking. These changes may affect the stability of Car_D2_^∙+^ relative to the other redox-active Car and Chl cofactors, because their redox potentials are so closely spaced.

It is known that there are at least two redox-active Car (Tracewell and Brudvig 2003; Telfer et al. 2003), and five redox-active Chl (Tracewell and Brudvig 2008) in the secondary electron-transfer pathways of PSII. However, the sequence of electron-transfer events and the specific identity of Car and Chl cofactors in the pathway are unknown (Faller et al. 2001). The effect of perturbing Car_D2_ on the rates and yields Chl^∙+^ and Car^∙+^ formation will depend on the connectivity of Car_D2_ with the other redox cofactors in the secondary electron-transfer pathway. For example, if another redox cofactor were capable of donating an electron to P_680_^∙+^ on an appropriate timescale, then the effect of perturbing Car_D2_ could be negligible. However, in each of the mutated PSII samples (D2-G47W, D2-G47F, and D2-T50F), a substantial decrease in yield of the secondary donors is observed by near-IR spectroscopy (Fig. 4A). Therefore, Car_D2_ seems to act as a bottleneck, resulting in decreased yield of the Car^∙^ peak at 750 nm, the Chl^∙+^ peak from 800 to 840 nm, and the Car^∙+^ peak near 1,000 nm in all mutated PSII samples. Thus, there is no efficient alternative pathway for transferring electrons to P_680_^∙+^. Similarly, as observed by EPR spectroscopy around the *g* = 2 region, the kinetics of formation for the secondary donor radicals are much slower in the G47F and G47W-mutated PSII samples than in the WT sample, although they are comparable to WT in the T50F-mutated PSII sample, which was modeled as having the smallest perturbation to Car_D2_ (Fig. 9). The G47F and G47W-mutated PSII samples are less efficient at forming a charge separation between Q_A_^−^ and the secondary donors, indicating that Car_D2_ is involved in this process. The decreased yield and impaired kinetics of the mutated PSII samples indicate that Car_D2_ is an early intermediate in secondary electron transfer, consistent with Car_D2_ being the initial electron donor to P_680_ and the initial step in an extended “branched” secondary electron-transfer pathway.

In addition to the decreased overall radical yield, there is a specific perturbation of the near-IR spectrum in each mutated PSII sample: the maximum of the Car^∙+^ peak is shifted to slightly longer wavelengths (Fig. 4B), while the maxima of the Chl^∙+^ and Car^∙^ peaks remain unchanged. This indicates that the Car^∙^ is not generated from Car_D2_, but most likely from a Car with a nearby proton accepting amino acid residue, as previously proposed (Gao et al. 2009). Furthermore, when the Car^∙+^ peak is deconvoluted into two Gaussian components, each corresponding to a redox-active Car^∙+^ (Tracewell and Brudvig 2003), the shorter-wavelength component shifts significantly more than the longer-wavelength component (more than three times, see Table 1). In WT PSII, the shorter-wavelength component has a maximum at 980 nm and a FWHM of 37.9 nm, and is the dominant contribution to the Car^∙+^ peak at 20 K. It decays much more quickly than the longer-wavelength component, although it remains the dominant contribution to the peak over 8 h of dark decay, indicating that it is closer to Q_A_^−^ than is the other Car^∙+^. We assign this faster-decaying, shorter-wavelength component with a maximum at 980 nm to Car_D2_^∙+^. Although Car_D2_ has been proposed to be the initial electron donor in the pathway of secondary electron transfer (Lakshmi et al. 2003; Tracewell and Brudvig 2003), the specific spectral perturbations of site-directed mutations near Car_D2_ on the 980 nm Car^∙+^ species provide the first direct evidence that Car_D2_ is one of the redox-active Car in PSII.

Previous studies have shown that the maximum of the Car^∙+^ near-IR peak shifts to a slightly shorter wavelength when Y_D_ is oxidized to Y_D_^∙^ in all PSII centers (Tracewell and Brudvig 2003). It was hypothesized that this was either due to an electrochromic shift caused by Y_D_ or due to biasing electron transfer so that the redox-active Car closest to Y_D_^∙^ would remain reduced to avoid electrostatic repulsion. However, it has been observed that electrochromic shifts propagate substantial distances through PSII. For example, generating Q_A_^−^ affects the visible spectrum of B_A_, the accessory Chl near P_A_ of P_680_, from 21 Å away, and also possibly affects the spectrum of B_B_, 29 Å away (Stewart et al. 2000). Although Y_D_^∙^ would most likely have a smaller electrochromic effect than Q_A_^–^, its effects do propagate at least as far as P_680_ (Diner and Rappaport 2002). Car_D2_ is approximately 25 Å from Y_D_. Alternatively, there are several Car cofactors in CP47 that are at a comparable or even shorter distance from Y_D_; one Car in CP47 is 21 Å from Y_D_, another is 27 Å away, and two others are about 30 Å from Y_D_. Due to closely spaced distances, an electrochromic shift would not be a definitive indicator of which Car is oxidized, even if it were observable at those distances. It is also possible that oxidation of Y_D_ may bias the path of secondary electron transfer. To pull an electron from one of the Car in CP47, two intermediate Chl^∙+^ would be involved that are each 20 Å from Y_D_^∙^, to ultimately generate a terminal Car^∙+^ that may be as close as 21 Å to Y_D_^∙^. Under these conditions, the 980 nm Car_D2_^∙+^ may be a more stable radical than the 999 nm Car^∙+^, resulting in a net shift of the Car^∙+^ peak to a shorter wavelengths.

The near-IR spectra of D2-G47W, D2-G47F, and D2-T50F PSII samples contain a relatively larger amount of the Chl^∙+^ peak as compared to the Car^∙+^ peak than WT PSII samples (Fig. 4B). One possibility is that the mutations around the headgroup of Car_D2_ caused a shift of the reduction potential of Car_D2_^∙+^ to a higher value, making it more difficult to oxidize Car_D2_ relative to other Chl and Car cofactors. This would destabilize Car_D2_^∙+^, which is the predominant donor in the charge separation (980 nm Car^∙+^, see Fig. 5; Table 1), thus favoring Chl^∙+^ in a greater portion of PSII centers. This model can explain the observations for the G47F PSII sample, which has both a lower yield of Car_D2_^∙+^ relative to the other Car^∙+^ and also a higher yield of Chl^∙+^. Alternatively, the mutations around the headgroup of Car_D2_ and its change in conformation may have affected the distances to other cofactors, biasing the electron-transfer pathway in a different direction, such as towards CP47, which is adjacent to the mutations and contains an extended cluster of Chl relatively close to Car_D2_ (Fig. 2). This model can explain the observations for the G47W PSII sample, which has the largest relative amount of Chl^∙+^ and also has the most Car_D2_^∙+^ compared to the other Car^∙+^. It is likely that a combination of these factors occurs. Regardless, the relative Chl^∙+^ radical yield is higher in each of the mutated PSII samples.

The mutated PSII samples isolated from cells grown at higher light exhibit a dark-stable radical observed by EPR spectroscopy (Fig. 7). The dark-stable radical has the appearance of an organic radical, and could be either a Chl^∙+^ or Car^∙+^, although it is unusual in that it persists on ice for more than 2 min in the dark. However, a similar observation has been made for PSII samples subjected to photoinhibitory illumination (Blubaugh et al. 1991). The G47F PSII sample has the largest amount of the dark-stable radical, and it also has the slowest kinetics of charge separation. Therefore, it is possible that the dark-stable radical is associated with a quenching state, such that there is a decrease in the stability and efficiency of charge separation (Schweitzer and Brudvig 1997).

In addition, the shape of the Chl^∙+^ peak appears to depend on the light exposure during growth. The PSII samples isolated from G47W cells grown at 10 μEinsteins/m^2^/s, and from T50F cells grown at 10 μEinsteins/m^2^/s show a double Chl^∙+^ peak with maxima at 812 and 826 nm. Conversely, PSII isolated from G47F cells grown at 40 μEinsteins/m^2^/s and from T50F cells grown at 40 μEinsteins/m^2^/s only display one Chl^∙+^ peak. Moreover, the G47F and T50F PSII samples from cells grown under 40 μEinsteins/m^2^/s of illumination contain the largest amounts of the dark-stable radical. This suggests that the dark-stable radical may reflect a bias in the pathways of secondary electron transfer such that fewer Chl cofactors are oxidized in PSII samples isolated from cells grown under high light than those grown under lower light conditions. The Chl^∙+^ peak in WT PSII also appears to have only one peak, but it is broader than the single peak in T50F and G47F PSII samples. It seems that the double Chl^∙+^ peak is observed for cells grown under lower light. A double Chl^∙+^ peak has been previously observed for spinach PSII, but not for *Synechocystis* PCC 6803 PSII (Tracewell et al. 2001). Perhaps the double versus single Chl^∙+^ peak correlates in some way with photodamage and/or photoprotection, rather than an intrinsic species difference.

The Car^∙+^ near-IR absorption peak is wider in the mutated PSII samples relative to WT PSII samples, an indication that the Car^∙+^ population may have become less homogeneous as a result of the mutations. The G47W and T50F PSII samples have the widest Car^∙+^ peaks (Fig. 4). These wider peaks may be an indication that more than one longer-wavelength Car^∙+^ contributes to the peak; because the longer-wavelength Car^∙+^ arise from a charge separation that is more stable than that involving Car_D2_^∙+^, they would include components that are located further from Q_A_^–^ than Car_D2_. Using high-frequency saturation-recovery EPR experiments, it has been found that the average distance from the nonheme iron to Car^∙+^ is 38 ± 1 Å (Lakshmi et al. 2003). Because Car_D2_^∙+^ is 36 Å from the nonheme iron, we can hypothesize that other candidate Car^∙+^ would be located about 40 Å from the nonheme iron. There are three Car molecules that are 40 Å from the nonheme iron: Car_D1_, a Car located at the interface of CP43 and PsbZ, and a Car located at the interface of CP47 and PsbM. There is previous evidence that Chl_ZD1_, which is adjacent to Car_D1_, can be oxidized (Stewart et al. 1998). Car_D1_ oxidation is also observed in isolated PSII reaction centers, containing the subunits D1, D2, Cyt *b*_559_, and PsbI (Telfer et al. 1991). However, the two Car located at interfaces 40 Å from the nonheme iron are further from Q_A_^–^, and would, therefore, recombine more slowly than Car_D2_^∙+^, and are also located near lipids that may have an affect on their redox potential (Tracewell and Brudvig 2008). More evidence is required to identify the precise location of the longer-wavelength absorbing Car^∙+^. However, the shorter-wavelength Car^∙+^ component, with a maximum at 980 nm in WT, is Car_D2_^∙+^, as indicated by the significant shift of its wavelength maximum following a mutation around the headgroup of Car_D2_.

## Electronic supplementary material

Below is the link to the electronic supplementary material.

Supplementary material 1 (PDF 189 kb)

## Abbreviations

β-DM: β-Dodecylmaltoside
Car: β-Carotene
Car^∙+^: Oxidized Car
Chl: Chlorophyll
Chl^∙+^: Oxidized Chl
CP47: 47 kDa Chlorophyll-binding protein of photosystem II
Cyt *b*_559_: Cytochrome *b*_559_
D1/D2: Homologous PSII reaction center core proteins
DCMU: 3-(3,4-Dichlorophenyl)-1,1-dimethylurea
EPR: Electron paramagnetic resonance
FWHM: Full width at half maximum
His-tagged PSII: *Synechocystis* PCC 6803 PSII containing hexa-histidine-tagged CP47
MES: 2-[*N*-morpholino] ethanesulfonic acid
OEC: Oxygen-evolving complex
P_680_: Primary chlorophyll electron donor in PSII
Pheo: Pheophytin
PSII: Photosystem II
PCR: Polymerase chain reaction
Q_A_: Non-exchanging plastoquinone electron acceptor in PSII
Q_B_: Exchangeable plastoquinone electron acceptor in PSII
WT: Wild type
Y_Z_/Y_D_: Redox-active tyrosines in the D1/D2 polypeptides
