# Supplementary material for: Using site-directed mutagenesis to probe the role of the D2 carotenoid in the secondary electron-transfer pathway of photosystem II
Source: Photosynth Res. 2013 Jan 21;120(1):141–52. doi: 10.1007/s11120-013-9793-6 (PMC3961632; doi:10.1007/s11120-013-9793-6)
Supplement: Supplementary file 1 — Supplementary material 1 (PDF 189 kb) [file 11120_2013_9793_MOESM1_ESM.pdf]

Supplementary Material

Using Site-Directed Mutagenesis to Probe the Role of the D2 Carotenoid in the Secondary Electron-Transfer Pathway of Photosystem II

Katherine E. Shinopoulos,[1] *Jianfeng Yu,*[2] *Peter J. Nixon*[2] *and Gary W. Brudvig**[,1]

[1]*Department of Chemistry, Yale University, New Haven, CT 06520-8107, USA and*
[2]*Division of Molecular Biosciences, Sir Ernst Chain Building – Wolfson Laboratories, Imperial College London, S. Kensington Campus, London, SW7 2AZ, UK.*

*To whom correspondence should be addressed.
E-mail, gary.brudvig@yale.edu, phone (203) 432-5202, fax (203) 432-6144

Contents:

Gaussian deconvolutions of the T50F and G47F PSII near-IR spectra at 0 and 3 hours of dark incubation

The Gaussian deconvolutions of the T50F and G47F PSII near-IR spectra at 0 and 3 hours of dark incubation are shown in Figure S1. They are analogous to the deconvolutions of the WT and G47W PSII near-IR spectra shown in Figure 5 of the main paper. The deconvolutions of the G47F near-IR spectra fit slightly less well than the others due to a lower concentration of Chl in the sample and a small signal in the raw data. The T50F displays a similar ratio of longer- and shorter-wavelength components as WT PSII.

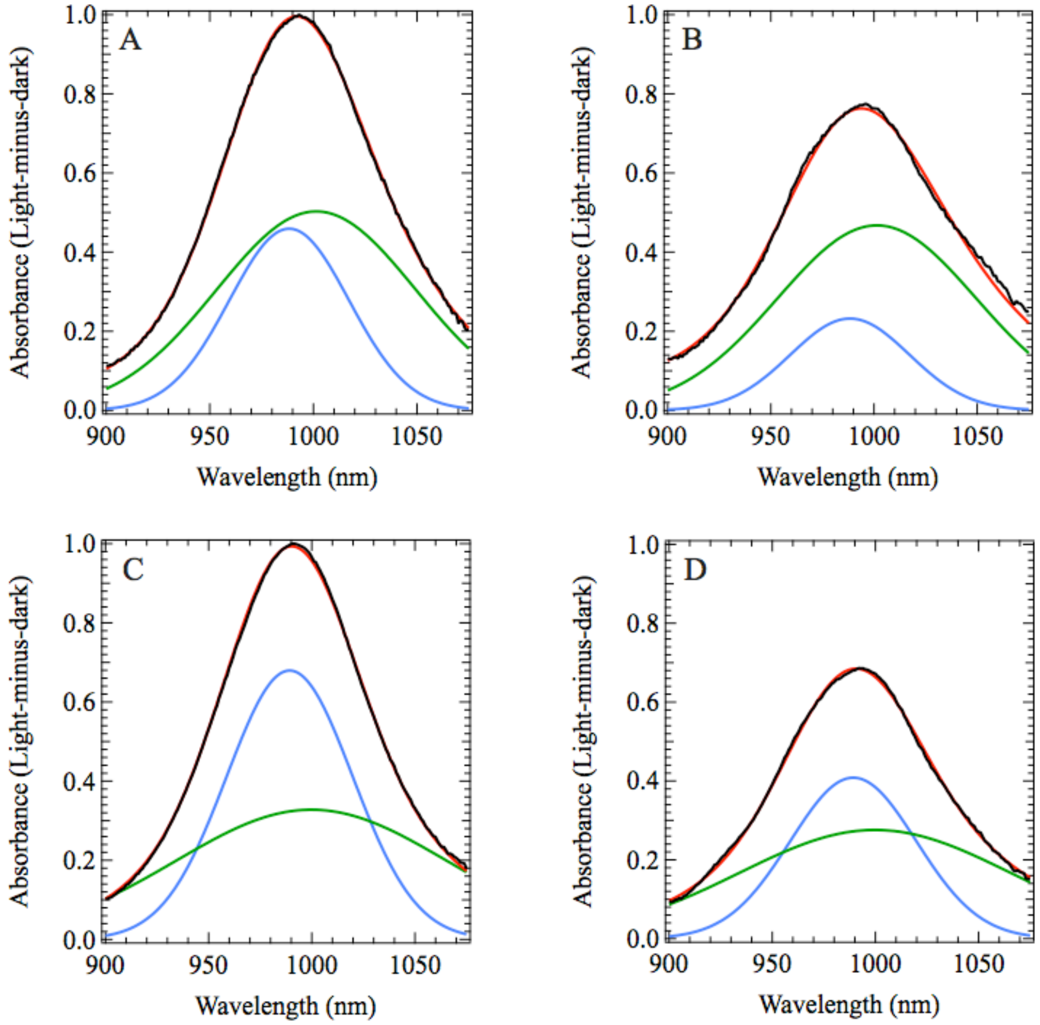

Fig. S1 Gaussian deconvolutions of A: the G47F PSII difference spectrum after 0 minutes of dark incubation, B: the G47F PSII difference spectrum after 3 hours of dark incubation, C: the T50F PSII difference spectrum after 0 minutes of dark incubation, and D: the T50F PSII difference spectrum after 3 hours of dark incubation. The two Gaussian components are shown in blue (shorter-wavelength component) and green (longer-wavelength component), their sum is shown in red, and the raw data are shown in black.
